# Perceived food palatability, blood glucose level and future discounting: Lack of evidence for blood glucose level’s impact on reward discounting

**DOI:** 10.1371/journal.pone.0255484

**Published:** 2021-08-09

**Authors:** Rafał Muda, Przemysław Sawicki, Michał Ginszt

**Affiliations:** 1 Faculty of Economics, Maria Curie-Sklodowska University, Lublin, Poland; 2 Centre for Economic Psychology and Decision Sciences, Kozminski University, Warsaw, Poland; 3 Department of Rehabilitation and Physiotherapy, Medical University of Lublin, Lublin, Poland; Universidad Loyola Andalucia Cordoba, SPAIN

## Abstract

Some previous studies have shown that an increase in blood glucose level makes people more future oriented, however, results are inconsistent, other studies failing to replicate this effect. Here, we tested whether psychological factors (in this instance, perception of food pleasantness after consumption of more palatable or less palatable meal) can play a moderating role. We hypothesized that consuming more palatable food (perceived as rewarding) should cause blood glucose levels to affect future discounting, but that this should not occur for the consumption of less palatable food. A high-powered, independent groups experiment (*N* = 149, power β = .90) showed that, subsequent to performing an initial discounting task, the two groups consuming a meal (a control group consumed no meal) displayed a significant increase in blood glucose levels 10 minutes after meal consumption and just before repeating the discounting task. However, the increased blood glucose levels did not cause changes in delay discounting in either experimental group.

## Introduction

Both media coverage and scientific studies have highlighted the fact that blood glucose plays a substantial role in fueling the human brain. Blood glucose has been connected with a wide range of behaviors and aspects of cognitive performance, e.g., memory [1], visuomotor task performance [2], and delay discounting [3, 4]. In the latter case, previous work shows that an increase in blood glucose level shifts people’s preferences toward being more future oriented [4, 5]. Simply put, a high level of blood glucose prompts greater patience, people preferring to wait for later larger rewards instead of taking sooner and smaller rewards [3–5]. However, recent literature on blood glucose levels and delay discounting has shown some inconsistency, papers appearing which show no effect [6, 7]. One recent paper [8] has proposed that blood glucose level’s effect on decision-making processes might be moderated by self-motivation factors, it being suggested that the pleasantness of a consumed meal is a rewarding experience which shifts people’s preferences [9]. The current work aimed to test this suggested mechanism. Support for this notion would open up the possibility of developing effective intervention methods to help people overcome impulsive behaviors resulting from a lack of patience.

People characterized by steeper discounting are at greater risk of substance abuse and obesity, and are more likely to engage in risky behaviors such as over-borrowing and risky sexual activity–for a review, see [10]. Steep delay discounting means that an individual would prefer to receive a sooner smaller reward instead of waiting for a larger later reward [11]. Because such maladaptive behaviors lead to both significant societal and economic costs [12, 13], researchers have attempted to find methods of reducing the steepness of delay discounting in people, and methods which have been found to be effective include: engaging in episodic future thinking [14, 15], spending time out of doors or generally cueing thoughts of nature settings (i.e., presenting nature photos prior to making delay discounting decisions) [16, 17], and, as previously mentioned, increasing people’s blood glucose levels [3–5].

A close look at studies of the effect of blood glucose on delay discounting reveals three strands of findings: (1) a first strand showing that increasing blood glucose levels via caloric intake serves as a metabolic cue providing an organism with information that it can afford to be more patient in waiting for subsequent rewards [3]; (2) a second strand showing that simply registering a sweet flavor in the mouth (without any caloric intake) can serve as a psychological factor that produces the same effect as increasing blood glucose level via food intake [18, 19]–this happens because sensing carbohydrates activates regions of the brain engaged in reward processing [20, 21]; (3) a third strand consisting of studies that have failed to replicate the finding that blood glucose levels have an effect on delay discounting [6–8].

In the current work, we attempted to combine findings from the above mentioned strands of research: we sought to test whether the effect of blood glucose on delay discounting is moderated by psychological factors. We hypothesized that food consumption (and in turn an increase in blood glucose level) would impact delay discounting when more palatable food–perceived as rewarding–was consumed (we operationalized this as a psychological factor), but that there would be no effect when less palatable food was consumed. This hypothesis was based on findings showing that food and monetary rewards are processed similarly in the brain [22], and that reward magnitude influences how our brains process the receipt of rewards [23]. We reasoned that the more palatable food should produce a signal strong enough to activate the reward system, and thus shift people’s preferences toward larger and later rewards, whereas the less palatable food should be less likely to be rewarding enough to have such an impact.

It should be noted that either soft drinks or glucose have been used to increase blood glucose levels in almost all previous studies. All of these are sweet and should also activate the motivational mechanisms potentially underlying the effect under examination, and in a study using the same procedure as previous studies, but in which a more complex meal was used (i.e., solid food with complex nutritional values), the effect of blood glucose on delay discounting was non-significant [8]. Thus, we believe that the proposed mechanism whereby psychological perceptions of food moderate the effect of blood glucose level on delay discounting has not been adequately tested previously.

## Materials and methods

### Participants

We recruited 155 participants through an external research company. The sample size was determined using sensitivity analysis, with *N* = 153 providing β = .90 power to detect an effect as small as *f* = 0.08 (*d* = 0.16), assuming a correlation among repeated measures of *r* = 0.85, three groups, and two measurements [24]. All participants were informed that the study was investigating the association between blood glucose levels and financial decision making, with real-life payoffs being conditional on their choices. All the participants were healthy individuals in the age range 18 to 35 years who were not on a diet excluding their consumption of the ingredients of the meals provided (e.g., they were not vegetarians or vegans). Seven participants were excluded before our main analysis because they did not meet the inclusion criteria relating to discounting task consistency, and it was therefore impossible to compute their discount parameters (see the delay discounting task section for more details). Thus, we analyzed data for 149 participants (*M*_age_ = 23.4 years, *SD*_age_ = 3.70 years; 67.8% females). Participants had an average weight of 66.9 kg (*SD* = 13.00), an average height of 171 cm (*SD* = 8.28), and an average BMI of 23.00 kg/m^2^ (*SD* = 3.39).

### Procedure

The study was carried out according to the Helsinki Declaration’s recommendations and with the consent of the Ethics Committee of the Kozminski University. During an online recruitment procedure (service done by Flow Research Center, Poland) participants were informed about the study’s objectives and asked to visit our laboratory on an empty stomach at least 6 hours after their last meal. We also instructed them not to eat or drink sweet beverages or alcohol the evening day before they participated in the experiment. The experiment commenced between 8 a.m. and 10 a.m. The whole procedure was run during one session. Participants began by providing written consent for their participation, and then anthropometric measures (height and weight) were taken. Next, the first measurement of blood glucose level was taken by a trained paramedic. A blood sample was taken from the middle, ring, or small finger of participants’ non-dominant hands using an Optium Xido blood glucose monitor (manufactured by Abbott Diabetes Care Inc., Alameda, CA, USA), in accordance with World Health Organization guidelines [25]. We collected capillary blood samples rather than venous blood since the former is more sensitive to glycaemic responses and displays lower between-subjects variation [26].

After their blood glucose level was measured, participants completed the discounting task for the first time. To increase their engagement, we informed them that they would have the opportunity to win a prize, which would be dependent on a choice they made: one randomly selected choice from both measurements would be realized in line with the characteristics of the alternative they chose. For example, if they opted for a larger later reward such as 200 PLN (about USD 50) in two weeks on the choice selected for payment, they received a 200 PLN payment two weeks after the experimental session. The payment was delivered to participants by a research assistant.

**Fig 1:**
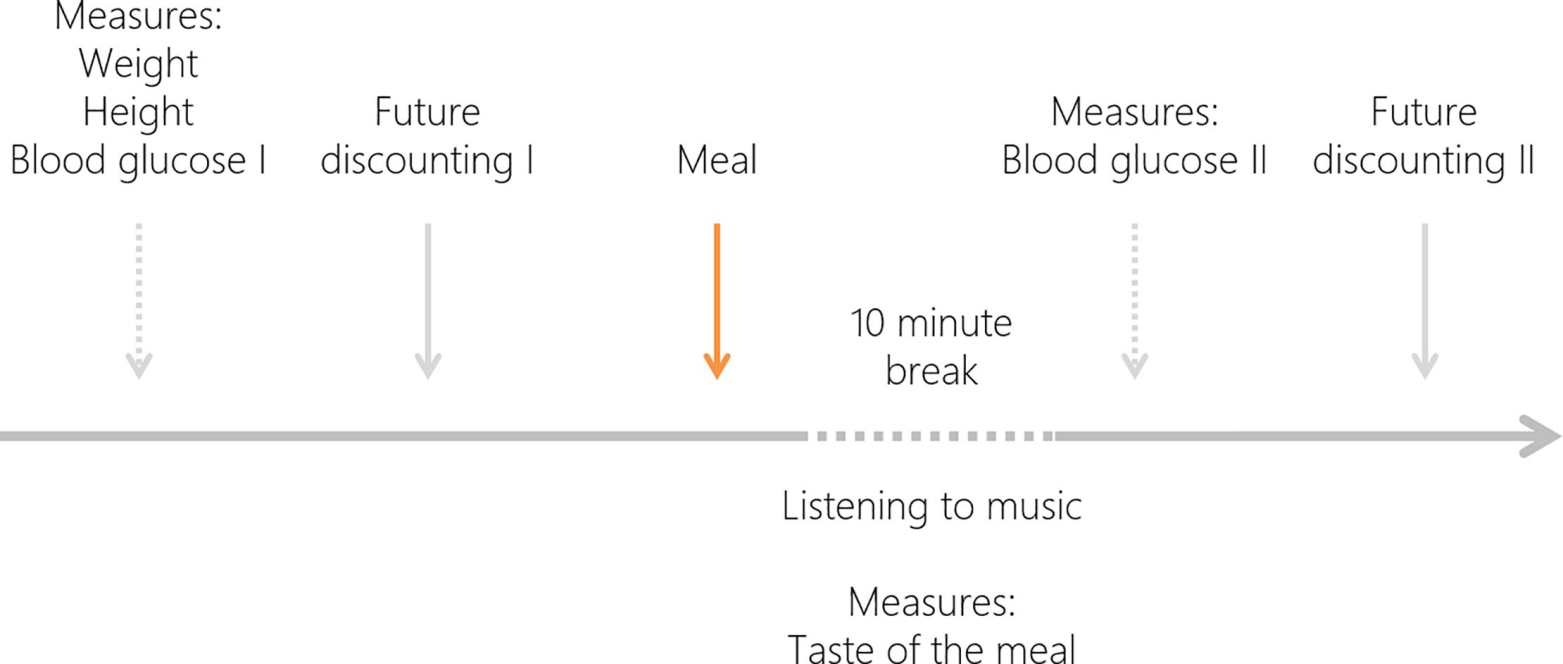
Timeline of the experimental procedure.

The discounting task was followed by a meal eating phase. Depending on their experimental condition (randomly selected for each participant), participants ate a more palatable meal, a less palatable meal (the taste of these meals was pre-tested, see S1 Appendix for more details), or did not receive a meal. After eating a meal, participants rated the taste of their meal (in line with the method proposed by Flint et al. [27] and Symmonds et al. [28], see S1 Appendix for the questionnaire used). Next, participants moved to a resting room for a 10 minute break, and had the opportunity to listen to music on a tablet computer using headphones. Subsequently, a second blood glucose measurement was taken, and participants performed the discounting task again (this was a repetition of the initial discounting task). The timeline of the experiment is presented in Fig 1.

#### The delay discounting task

To measure future discounting at both time points, participants completed the Monetary Choice Questionnaire (MCQ) [29]. This questionnaire consists of 27 choices between smaller sooner and larger later payoffs in the domain of gains (e.g., $69 “now” or $85 in 91 days). For each choice, a discount-rate parameter *k* can be estimated. The MCQ characterizes respondents using 9 groups of different discount-rate parameters (i.e., for each group, three choices have the same *k* value but differ in their payoffs and delays, e.g., the trials “$19 now or $25 in 53 days”, “$40 now or $55 in 62 days”, and “$55 now or $75 in 61 days”, all have a *k* value of 0.006). To correctly estimate a discount-rate parameter for a person, a certain amount of consistency among preferences is necessary, i.e., for a specific group of choices a person should consistently select smaller sooner options or consistently select larger later options. If consistency is lower than 80% it is impossible to estimate a person’s discount-rate parameter and it is necessary to exclude their data from analysis [30].

#### Meals

Two types of meal were prepared so that they had different sensory profiles (a more palatable meal and a less palatable meal). Both meals were identical in appearance (the meal itself, the type of plate, and the cutlery supplied), main food ingredients, total calories (550 kcal), and the percentage of calories contributed by different nutritional contents (carbohydrates 55%, fat 20%, protein 25%). The only difference was in the way the meals were prepared and the condiments that were used: the less palatable meal had almost no salt and pepper added, contained non-juicy dry meat and burnt sun-dried tomatoes (which made the tomatoes taste bitter), and had overcooked potato dumpling. All these features were intended to make the less palatable meal less pleasant. The meals were prepared by the same experienced cook immediately prior to consumption. All participants given a meal received a glass of still water (250 ml) with their meal. A researcher was present during consumption of the meal to ensure compliance with procedural requirements. The entire meal was consumed within a time period of between 5 and 15 minutes.

### Statistical analyses

2x3 ANOVA with the first factor referring to time of measurement (pre-manipulation vs. post-manipulation; within-subject) and the second factor referring to consumed meal (more palatable meal vs. less palatable meal vs. no meal; between-subject) was used to assess changes in: (1) blood glucose level, and (2) participants’ discount parameters.

To test whether the experimental manipulation was successful (in terms of experimental group participants’ perceptions of their meal’s pleasantness), we computed a mean value from ratings of smell, taste, aftertaste, and palatability, and compared the resulting values using an independent samples *t*-test with meal (more palatable meal vs. less palatable meal) as a grouping variable.

All post-hoc tests were Bonferroni corrected.

Data and code are to be found: https://osf.io/5fnwr/

## Results

### The experimental manipulation’s effect on perceptions of meals’ pleasantness

**Fig 2:**
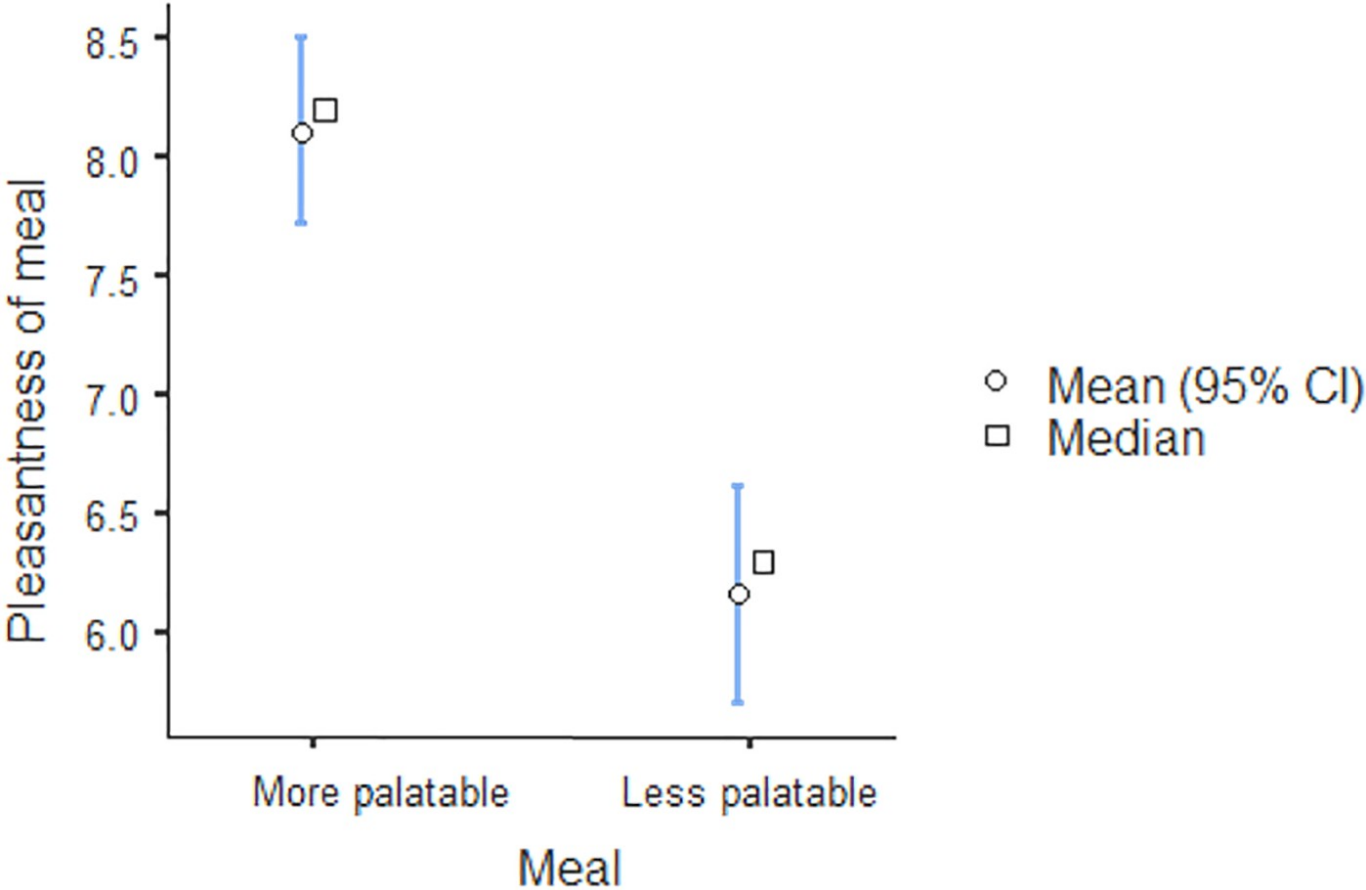
Pleasantness of meals according to experimental condition.

As predicted, we observed a significant difference in perceptions of the pleasantness of the meals served, *t*(101) = 6.55, *p* < .001, *d* = 1.30 [0.82, 1.77]: on a scale from 0 to 10, participants in the more palatable meal condition rated their meal as highly pleasant (*M* = 8.09, *SD* = 1.45), whereas participants in the less palatable meal condition only rated their meal’s pleasantness as moderate (*M* = 5.77, *SD* = 2.00 –see Fig 2 for more details). Thus, we concluded that our experimental manipulation was highly successful.

### Blood glucose levels

**Fig 3:**
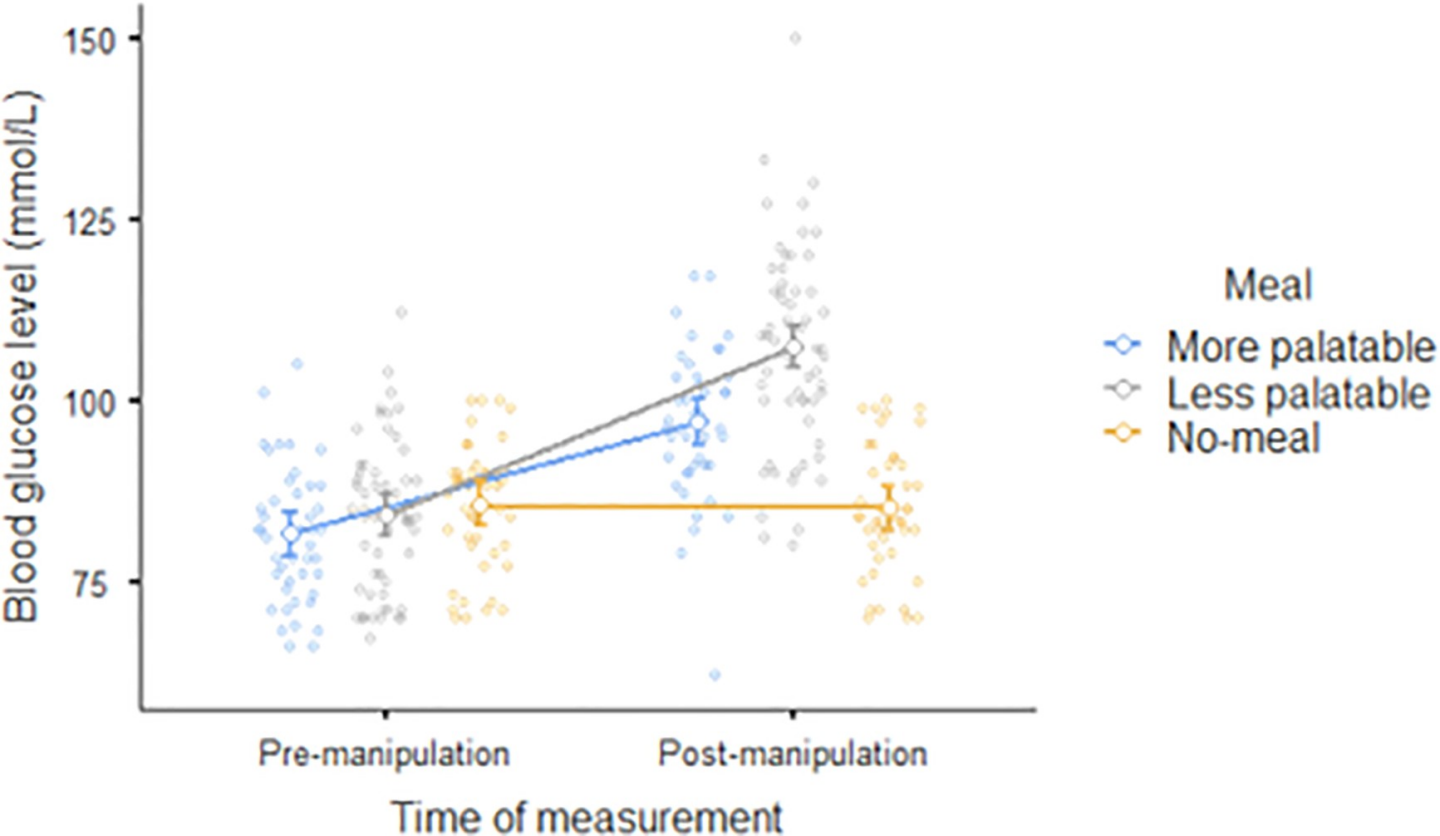
Mean pre-manipulation and post-manipulation levels of blood glucose for three groups varying in meal consumption. Dots indicate observed scores.

The ANOVA for blood glucose levels revealed significant main effects for both time of measurement, *F*(1, 145) = 163.1, *p* < .001, η_p_^2^ = 0.529, and meal, *F*(2, 145) = 18.5, *p* < .001, η_p_^2^ = 0.203. There was also a significant time of measurement x meal interaction, *F*(2, 145) = 49.8, *p* < .001, η_p_^2^ = 0.407. Decomposing the interaction, we observed increases in blood glucose after consuming meals for both the group given the more palatable meal, *t*(145) = 8.63, *p* < .001, and the group given the less palatable meal, *t*(145) = 14.67, *p* < .001, but not for the group given no meal, *t*(145) = -0.27, *p* > .999. These results also suggested that the experimental manipulation was robust. Detailed results are presented in Fig 3.

### Individual discount parameters

Regarding this analysis, we extended the classical NHST analysis (based on p-vales) with Bayesian analysis. Bayesian analysis allow not only to accept the alternative hypothesis but also to support null effects. Specifically, Bayes factor weights evidence supporting null and explicitly stated hypothesis giving the precise ratio of support. For example, B_10_ = 10 suggests that alternative hypothesis is 10 times more likely than the null, given the data collected. Or that 10 out of 11 times such results would be obtained given H1 being true. Prior to analysis, we estimated participants’ discount parameters with the dedicated auto-scorer provided by Kaplan and colleagues [30], which returns natural log *k* values fitted to a hyperbolic curve. Lower log *k* values indicate less-steeper discounting of future rewards. ANOVA results revealed only a significant main effect of time of measurement, *F*(1, 146) = 5.57, *p* = .020, η_p_^2^ = 0.037, BF_M_ = 5.54, BF_10_ = 2.23. Participants showed steeper discounting of future rewards when performing the discounting task for the second time (see Table 1 for more details). Neither the main effect of meal, *F*(2, 146) = 0.36, *p* = .700, η_p_^2^ = .005, BF_10_ = 0.17, BF_exclusion_ = 7.95, nor the time of measurement x meal interaction, *F*(2, 146) = 0.54, *p* = .586, η_p_^2^ = 0.007, BF_10_ = 0.40, BF_exclusion_ = 22.27 was significant. Results of Bayesian analysis (run with default priors) showed that the best model included only effect of time of measurement. Inclusion of meal effect and meal x time of measurement interaction into the model provided evidence against the idea that increase in blood glucose level nor pleasantness of the meal consumed impacted discount rates. Note that BF_exclusion_ = 22.27 means that our data changed the odds in favor of models that do not include the specific predictor by a factor of 22.

**Table 1:** Mean levels of participants’ discount parameters (log *k*) for pre-manipulation and post-manipulation measurements. Lower values indicate steeper discounting of future rewards.

				95% Confidence Interval	
Meal	Time of measurement	Mean	*SE*	Lower	Upper
More palatable	Pre-manipulation	-1.96	0.0996	-2.16	-1.77
	Post-manipulation	-2.01	0.0996	-2.20	-1.81
Less palatable	Pre-manipulation	-1.88	0.0943	-2.07	-1.70
	Post-manipulation	-2.04	0.0943	-2.23	-1.86
No-meal	Pre-manipulation	-1.82	0.0991	-2.02	-1.62
	Post-manipulation	-1.94	0.0991	-2.14	-1.75

## Discussion

We observed no evidence that increases in blood glucose levels lead to changes in future discounting of monetary rewards. This was true both for meals perceived as highly pleasant and for meals perceived as only moderately pleasant. In general, people consuming meals made the same decisions as those not consuming meals, whose blood glucose levels were not changed. We observed a decrease in strength of future discounting among all three groups: participants slightly changed their preferences toward larger later payoffs after 10 minutes waiting time. These results provide evidence that increasing people’s blood glucose levels using food with complex nutritional contents (as opposed to sweet beverages) does not influence future discounting levels [8]. In a more general sense, the results also provide additional evidence about the lack of a connection between blood glucose levels and discounting of monetary rewards, and confirm some other previous findings also showing that increases in blood glucose levels do not impact delay discounting [6–8].

Our findings add various insights to the debate as to whether either energy factors are solely responsible for changes in delay discounting (e.g., food ingestion, [5, 31]) or whether motivational aspects also play a part (e.g., as tested in studies where people rinse their mouths with a sugared beverage without swallowing it [18, 19]). In a recent paper [8] in which no changes in delay discounting were observed even in the presence of extremely large increases in blood glucose levels, it was theorized that behavioral responses to increases in levels of blood glucose might be conditional on the type of meal consumed. More specifically, it was suggested that the food consumed needs to be desirable and produce rewarding feelings. Simply put, the food needs to be tasty and perceived as something more pleasant than the usual homemade breakfast. In our experiment, we manipulated the taste of meals very successfully while keeping their caloric and nutritional values at the same level in both the more palatable and less palatable meal groups. If the hypothesis suggesting a mediating role for the pleasantness of a meal is true, people given a tasty meal should be more patient (i.e., prefer larger later rewards), whereas people given a less tasty meal should not change their preferences. But this was not the case. Instead, we observed no difference between the relevant groups. The current results suggest either that the previously proposed mechanism might not play as significant a role as supposed, or that more rewarding food is needed, e.g., a hamburger or pizza. The need for different kind of food might be highly plausible explanation irrespectively of observed differences in perception of pleasantness of consumed meal between meal-groups–the more pleasant meal was rated on more than 8 on a 10 point scale, but this was a dinner-type meal. On a reflective level such a meal might be perceived as highly pleasant. On the deeper level, such a food rather does not produce crave to eat it once again or will not lead to overeating (in contrast to fast foods that might create addiction [32]). As we collected only self-rated measures, we do not have data to confirm above explanation. This would require neuro-imagining study what we perceive as interesting avenue for further research.

Another possible explanation for the present observation is that only sweet beverages cause blood glucose levels to have an effect on delay discounting: solid foods do not produce the same effect. To date, almost all studies have increased blood glucose levels by either using soft drinks with sugar or glucose itself [3, 5–7, 31]. To our best knowledge, only one previous study has used solid food, and this found no effect of blood glucose levels on delay discounting in the two experiments performed [8]. These differences may be attributable to differences in glucose responses between solid and liquid foods with comparable energy densities [33]. We cannot definitely state whether this is true on the basis of our data, but this possibility is a promising avenue for future research.

It is also possible that blood glucose levels did not influence delay discounting because of the task we used: delay discounting in a non-food domain was considered. As a recent meta-analysis on risk taking and blood glucose levels shows, low levels of blood glucose increase willingness to take risks, but only with respect to food: the effect does not transfer to non-food-related activities such as gambling [34]. This would suggest that low levels of blood glucose (i.e., hunger) should change people’s preferences toward smaller sooner food rewards, but that they should not affect preferences in the domain of monetary rewards. Future studies are needed to confirm this reasoning.

Finally, we should stress that we are not claiming that blood glucose does not impact delay discounting. In this paper, we have reported the results of only one experiment, and, although the experiment was highly-powered and adopted virtually the same procedures as those used in previous studies of blood glucose and delay discounting (see e.g., [3]), there are still questions to be answered and potential moderators to be tested (e.g. previously mentioned fast foods).

To summarize, we aimed to test whether food pleasantness moderates the relationship between people’s blood glucose levels and delay discounting. We successfully manipulated perceptions of meal pleasantness while controlling for caloric and nutritional values. We also successfully increased the blood glucose levels of two groups of participants consuming meals, but this did not lead to changes in delay discounting levels in either of these groups–there are several possible reasons for this observation which should be considered in future1studies.

## Supporting information

S1 Appendix Appendix glucose paper. (DOCX)

S1 Data (RAR)

## References

[1] Messier C. Glucose improvement of memory: a review. Eur J Pharmacol. 2004;490(1–3):33–57. doi: 10.1016/j.ejphar.2004.02.043 15094072

[2] Kaplan RJ, Greenwood CE, Winocur G, Wolever TM. Cognitive performance is associated with glucose regulation in healthy elderly persons and can be enhanced with glucose and dietary carbohydrates. Am J Clin Nutr. 2000;72(3):825–36. doi: 10.1093/ajcn/72.3.825 10966906

[3] Wang XT, Dvorak RD. Sweet future: fluctuating blood glucose levels affect future discounting. Psychol Sci. 2010;21(2):183–8. doi: 10.1177/0956797609358096 20424042

[4] Wang XTX-T, Huangfu G. Glucose-specific signaling effects on delay discounting in intertemporal choice. Physiol Behav. 2017;169:195–201. doi: 10.1016/j.physbeh.2016.12.001 27940144

[5] Wang XT, Reed RN, Baugh LA, Fercho KA. Resource forecasting: Differential effects of glucose taste and ingestion on delay discounting and self-control. Appetite. 2018;121:101–10. doi: 10.1016/j.appet.2017.11.083 29127026

[6] Lange F, Eggert F. Sweet delusion. Glucose drinks fail to counteract ego depletion. Appetite. 2014;75:54–63. doi: 10.1016/j.appet.2013.12.020 24389240

[7] Kuhn M, Kuhn P, Villeval MC. Self control and inter temporal choice; evidence from glucose and depletion interventions[C]. CESifo Work Pap Ser. 2014;4609.

[8] Sawicki P, Muda R, Goral K, Skrzypek M, Wiśniewska K, Bieniak M et al. Increasing blood glucose level via breakfast meals is not connected with changes in delay discounting. Physiology & Behavior. 2019;210:112619. 31323294 10.1016/j.physbeh.2019.112619

[9] Hammersley R, Reid M. Theorising transient mood after ingestion. Neurosci Biobehav Rev. 2009;33(3):213–22. doi: 10.1016/j.neubiorev.2008.07.010 18775746

[10] Rung JM, Madden GJ. Experimental reductions of delay discounting and impulsive choice: A systematic review and meta-analysis. J Exp Psychol Gen. 2018;147(9):1349–81. doi: 10.1037/xge0000462 30148386 PMC6112163

[11] Frederick S, Loewenstein G, O’Donoghue T. Time Discounting and Time Preference: A Critical Review. J Econ Lit. 2002;40(2):351–401.

[12] Wang YC, McPherson K, Marsh T, Gortmaker SL, Brown M. Health and economic burden of the projected obesity trends in the USA and the UK. Lancet Lond Engl. 2011;378(9793):815–25. doi: 10.1016/S0140-6736(11)60814-3 21872750

[13] Cawley J, Meyerhoefer C. The medical care costs of obesity: an instrumental variables approach. J Health Econ. 2012;31(1):219–30. doi: 10.1016/j.jhealeco.2011.10.003 22094013

[14] Daniel TO, Stanton CM, Epstein LH. The Future Is Now: Reducing Impulsivity and Energy Intake Using Episodic Future Thinking. Psychol Sci. 2013;24(11):2339–42. doi: 10.1177/0956797613488780 24022653 PMC4049444

[15] Liu L, Feng T, Chen J, Li H. The value of emotion: how does episodic prospection modulate delay discounting?. PLoS One. 2013;8(11):e81717. doi: 10.1371/journal.pone.0081717 24312341 PMC3842935

[16] Berry MS, Sweeney MM, Morath J, Odum AL, Jordan KE. The nature of impulsivity: visual exposure to natural environments decreases impulsive decision-making in a delay discounting task. PloS One. 2014;9(5):e97915. doi: 10.1371/journal.pone.0097915 24841421 PMC4026519

[17] van der Wal AJ, Schade HM, Krabbendam L, van Vugt M. Do natural landscapes reduce future discounting in humans? Proc Biol Sci. 2013;280(1773):20132295. doi: 10.1098/rspb.2013.2295 24197412 PMC3826228

[18] Sanders MA, Shirk SD, Burgin CJ, Martin LL. The gargle effect: rinsing the mouth with glucose enhances self-control. Psychol Sci. 2012;23(12):1470–2. doi: 10.1177/0956797612450034 23090756

[19] Molden DC, Hui CM, Scholer AA, Meier BP, Noreen EE, D’Agostino PR, i in. Motivational versus metabolic effects of carbohydrates on self-control. Psychol Sci. 2012;23(10):1137–44. doi: 10.1177/0956797612439069 22972907

[20] Kringelbach ML. Food for thought: hedonic experience beyond homeostasis in the human brain. Neuroscience. 2004;126(4):807–19. doi: 10.1016/j.neuroscience.2004.04.035 15207316

[21] Chambers ES, Bridge MW, Jones DA. Carbohydrate sensing in the human mouth: effects on exercise performance and brain activity. J Physiol. 2009;587(Pt 8):1779–94. doi: 10.1113/jphysiol.2008.164285 19237430 PMC2683964

[22] Simon JJ, Skunde M, Wu M, Schnell K, Herpertz SC, Bendszus M, i in. Neural dissociation of food- and money-related reward processing using an abstract incentive delay task. Soc Cogn Affect Neurosci. 2015;10(8):1113–20. doi: 10.1093/scan/nsu162 25552570 PMC4526483

[23] Goyer JP, Woldorff MG, Huettel SA. Rapid electrophysiological brain responses are influenced by both valence and magnitude of monetary rewards. J Cogn Neurosci. 2008;20(11):2058–69. doi: 10.1162/jocn.2008.20134 18416673 PMC3612129

[24] Faul F, Erdfelder E, Lang A-G, Buchner A. G*Power 3: a flexible statistical power analysis program for the social, behavioral, and biomedical sciences. Behav Res Methods. 2007;39(2):175–91. doi: 10.3758/bf03193146 17695343

[25] WHO Guidelines on Drawing Blood: Best Practices in Phlebotomy. Geneva: World Health Organization; 2010. 7, Capillary sampling. Available from: https://www.ncbi.nlm.nih.gov/books/NBK138654/23741774

[26] Brouns F, Bjorck I, Frayn KN, Gibbs AL, Lang V, Slama G, i in. Glycaemic index methodology. Nutr Res Rev. 2005;18(1):145–71. doi: 10.1079/NRR2005100 19079901

[27] Flint A, Raben A, Blundell JE, Astrup A. Reproducibility, power and validity of visual analogue scales in assessment of appetite sensations in single test meal studies. Int J Obes. 2000;24(1):38–48. doi: 10.1038/sj.ijo.0801083 10702749

[28] Symmonds M, Emmanuel JJ, Drew ME, Batterham RL, Dolan RJ. Metabolic state alters economic decision making under risk in humans. PLoS One. 2010;5(6):e11090. doi: 10.1371/journal.pone.0011090 20585383 PMC2886827

[29] Kirby K, Petry N, Bickel W. Heroin Addicts Have Higher Discount Rates for Delayed Rewards Than Non-Drug-Using Controls. J Exp Psychol Gen. 1999;128(1):78–87. doi: 10.1037//0096-3445.128.1.78 10100392

[30] Kaplan BA, Lemley SM, Reed DD, Jarmolowicz D P. 21- and 27-item monetary choice questionnaire automated scorers [software]. Lawrence, KS: Center for Applied Neuroeconomics, University of Kansas. 2014a. Available from http://hdl.handle.net/1808/15424.

[31] Wang X-T. Resource Signaling via Blood Glucose in Embodied Decision Making. Front Psychol. 2018;9:1965. doi: 10.3389/fpsyg.2018.01965 30374322 PMC6196271

[32] Garber A, H Lustig R. Is fast food addictive?. Current drug abuse reviews, 2011;4(3), 146–62. doi: 10.2174/1874473711104030146 21999689

[33] Shafaeizadeh S, Muhardi L, Henry CJ, van de Heijning BJM, van der Beek EM. Macronutrient Composition and Food Form Affect Glucose and Insulin Responses in Humans. Nutrients. 2018;10(2):188. doi: 10.3390/nu10020188 29419785 PMC5852764

[34] Orquin JL, Dalgaard Christensen J, Lagerkvist C-J. A meta-analytical and experimental examination of blood glucose effects on decision making under risk. Judgment and Decision Making. 2020;15(6):1024–36.

