# Supplementary material for: Perceived food palatability, blood glucose level and future discounting: Lack of evidence for blood glucose level’s impact on reward discounting
Source: PLoS One. 2021 Aug 9;16(8):e0255484. doi: 10.1371/journal.pone.0255484 (PMC8351949; doi:10.1371/journal.pone.0255484)
Supplement: S1 Appendix — (DOCX) [file pone.0255484.s001.docx]

S1 APPENDIX

S1 Table. Time discounting task

| **Trial ID** | **SS (in PLN)** | **SS payment time** | **LL (in PLN)** | **LL payment time** |
| --- | --- | --- | --- | --- |
| 1 | 54 | now | 55 | 117 days from now |
| 2 | 55 | now | 75 | 61 days from now |
| 3 | 19 | now | 25 | 53 days from now |
| 4 | 31 | now | 85 | 7 days from now |
| 5 | 14 | now | 25 | 19 days from now |
| 6 | 47 | now | 50 | 160 days from now |
| 7 | 15 | now | 35 | 13 days from now |
| 8 | 25 | now | 60 | 14 days from now |
| 9 | 78 | now | 80 | 162 days from now |
| 10 | 40 | now | 55 | 62 days from now |
| 11 | 11 | now | 30 | 7 days from now |
| 12 | 67 | now | 75 | 119 days from now |
| 13 | 34 | now | 35 | 186 days from now |
| 14 | 27 | now | 50 | 21 days from now |
| 15 | 69 | now | 85 | 91 days from now |
| 16 | 49 | now | 60 | 89 days from now |
| 17 | 80 | now | 85 | 157 days from now |
| 18 | 24 | now | 35 | 29 days from now |
| 19 | 33 | now | 80 | 14 days from now |
| 20 | 28 | now | 30 | 179 days from now |
| 21 | 34 | now | 50 | 30 days from now |
| 22 | 25 | now | 30 | 80 days from now |
| 23 | 41 | now | 75 | 20 days from now |
| 24 | 54 | now | 60 | 111 days from now |
| 25 | 54 | now | 80 | 30 days from now |
| 26 | 22 | now | 25 | 136 days from now |
| 27 | 20 | now | 55 | 7 days from now |

S2 Figure. Distribution of sum of larger and later choices for each condition. Section A presents results for first measurement. Section B for second measurement.

A


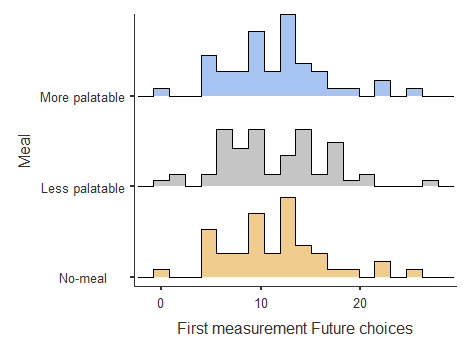


B


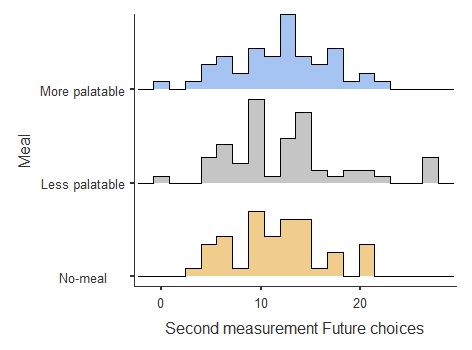


S3 Figure. Results of delay discounting task for pre- and post-manipulation measurement


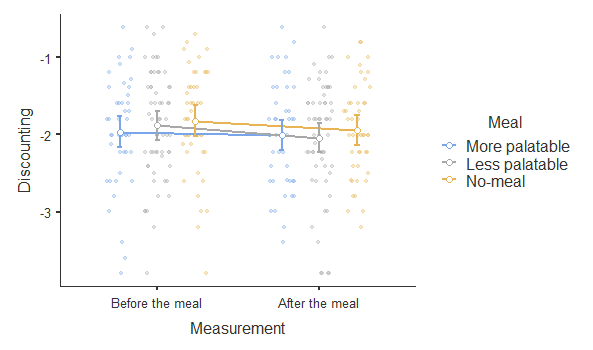


S4 Table. Post-hoc analysis for pre- and post-manipulation discounting task with type of the meal as grouping variable.

| **Measurement** | | **Meal** | |  | | **Measurement** | | **Meal** | | **Mean Difference** | | **df** | | **t** | | **p_bonferroni_** | | |  |
| --- | --- | --- | --- | --- | --- | --- | --- | --- | --- | --- | --- | --- | --- | --- | --- | --- | --- | --- | --- |
| Before the meal |  | More palatable |  | - |  | Before the meal |  | Less palatable |  | -0.0792 |  |  | 203 |  | -0.582 |  | > .999 |  | |
|  |  |  |  | - |  | Before the meal |  | No-meal |  | -0.1426 |  |  | 203 |  | -0.992 |  | > .999 |  | |
|  |  |  |  | - |  | After the meal |  | More palatable |  | 0.0454 |  |  | 146 |  | 0.541 |  | > .999 |  | |
|  |  |  |  | - |  | After the meal |  | Less palatable |  | 0.0814 |  |  | 203 |  | 0.598 |  | > .999 |  | |
|  |  |  |  | - |  | After the meal |  | No-meal |  | -0.0197 |  |  | 203 |  | -0.137 |  | > .999 |  | |
|  |  | Less palatable |  | - |  | Before the meal |  | No-meal |  | -0.0634 |  |  | 203 |  | -0.468 |  | > .999 |  | |
|  |  |  |  | - |  | After the meal |  | More palatable |  | 0.1246 |  |  | 203 |  | 0.915 |  | > .999 |  | |
|  |  |  |  | - |  | After the meal |  | Less palatable |  | 0.1606 |  |  | 146 |  | 2.170 |  | 0.474 |  | |
|  |  |  |  | - |  | After the meal |  | No-meal |  | 0.0595 |  |  | 203 |  | 0.440 |  | > .999 |  | |
|  |  | No-meal |  | - |  | After the meal |  | More palatable |  | 0.1880 |  |  | 203 |  | 1.308 |  | > .999 |  | |
|  |  |  |  | - |  | After the meal |  | Less palatable |  | 0.2240 |  |  | 203 |  | 1.655 |  | > .999 |  | |
|  |  |  |  | - |  | After the meal |  | No-meal |  | 0.1229 |  |  | 146 |  | 1.479 |  | > .999 |  | |
| After the meal |  | More palatable |  | - |  | After the meal |  | Less palatable |  | 0.0360 |  |  | 203 |  | 0.264 |  | > .999 |  | |
|  |  |  |  | - |  | After the meal |  | No-meal |  | -0.0651 |  |  | 203 |  | -0.453 |  | > .999 |  | |
|  |  | Less palatable |  | - |  | After the meal |  | No-meal |  | -0.1011 |  |  | 203 |  | -0.747 |  | > .999 |  | |

S5. Questions about the palatability of the consumed meal

| Bad | Visual appeal  _____________________________ | Good |
| --- | --- | --- |
| Bad | Smell  _____________________________ | Good |
| Bad | Taste  _____________________________ | Good |
| Bad | Aftertaste _____________________________ | Good |
| Bad | Palatability _____________________________ | Good |
